# Prevalence of Pulp Stones in Patients Visiting the Dental Hospital of Imam Abdulrahman Bin Faisal University: A Correlative Retrospective Study

**DOI:** 10.7759/cureus.77765

**Published:** 2025-01-21

**Authors:** Arishiya T Fairozekhan, Syed Z Ahmed, Faraz Mohammed, Abdul Khabeer, Hawra M Al Hamad

**Affiliations:** 1 Biomedical Dental Sciences, College of Dentistry, Imam Abdulrahman Bin Faisal University, Dammam, SAU; 2 Restorative Dental Sciences, College of Dentistry, Imam Abdulrahman Bin Faisal University, Dammam, SAU; 3 Medical Education, Schulich School of Medicine and Dentistry, University of Western Ontario, London, CAN; 4 College of Dentistry, Riyadh Elm University, Riyadh, SAU

**Keywords:** demographics, dental radiographs, medical conditions, medically compromised, prevalence, pulp stones, systemic diseases

## Abstract

Introduction: Dental pulp stones are distinct calcified bodies that can be found in teeth that are healthy, diseased, or even unerupted. Pulp stones are suggested to be a manifestation of various systemic and genetic diseases affecting different organs of the body. Therefore, this study aimed to correlate the prevalence of pulp stones with gender, nationality, age, dental status, and systemic diseases.

Methods: The medical records and radiographs of patients who visited the screening clinics and the Department of Oral Diagnosis at the College of Dentistry, Imam Abdulrahman Bin Faisal University, Dammam, Saudi Arabia, between January 2017 and June 2018 were analyzed in this study. Two examiners evaluated the digital orthopantomographs (OPGs) to identify the prevalence of pulp stones concerning the patient’s age, gender, nationality, arch position, and medical condition.

Results: A total of 153 patient records were examined, and pulp stones were detected in 43.1% of the patients. Among the nationalities, Saudi patients were the most affected at 57.6%, while 42.4% were non-Saudi. The maximum occurrence of pulp stones was observed in age group 4 (9.2%), while the minimum occurrence was in age group 8 (0.7%). The maximum occurrence of pulp stones (21.2%) was observed in age group 4 (36-45 years), while the minimum occurrence was 7.6% in age group 2 (16-25 years). Out of all examined patients, 46 (30.1%) patients were medically compromised. Among these medically compromised patients, radiographic examination showed that 56.5% (n=26) had pulp stones.

Conclusion: This study supports the idea that dental radiographs are useful in detecting pulp stones. Further research is required to explore the potential of using dental radiographs as a screening tool for the early detection of systemic diseases.

## Introduction

Pulp stones are distinct dental pulp calcified bodies that have a dentin-like ratio of calcium phosphorous and can be seen in teeth that are healthy, diseased, or even unerupted [1-3]. These pulp stones, also known as denticles, can exist as free, attached, or embedded calcified bodies within the pulpal tissue [4]. It has been reported that a tooth can possess between one and 12 stones, with dimensions ranging from tiny particles to massive masses occluding the pulpal space [5].

Based on the microscopic properties, the pulp stones have been categorized as true and false pulp stones [6]. True pulp stones are formed from epithelial-mesenchymal interactions, while false pulp stones are the result of degenerating pulpal cells that ultimately become mineralized [7]. True pulp stones have a tubular structure resembling dentin, while false pulp stones are concentric rings of calcified material not resembling dentin [8]. Other etiological factors involved in the formation of pulp stones include increased age, pulpal circulatory disturbances, orthodontic tooth movements, dental transplantations, and trauma [3,8]. Long-standing irritants, such as caries, large dental restorations, and chronic inflammation of the pulpal tissue, may be associated with their development, while other studies have suggested that the pulp stones are formed as a result when an irritated pulp tries to undergo repair [3].

Unless they invade any nerve fiber bundles, pulp stone is typically asymptomatic [9]. There are occasions when pulp calcification [PC) and pulp stones are linked to idiopathic pain. During root canal therapy, the pulp stones provide significant challenges. If they are found along the root curvature, they result in severe occlusion during the endodontic procedure [9]. Based on the radiographic analysis, the prevalence of pulp stones in teeth is estimated to be about 20-25%, although histological analyses indicate higher percentages [3]. Pulp stones appear as radiopaque calcified bodies, which often act as a hindrance during endodontic treatment [4]. A higher prevalence was found in the Saudi population (56.2%) [10].

Multiple studies have reported that pulp stones are a manifestation of systemic and genetic diseases affecting various organs of the body, leading to pathological biomineralization, such as hypercalcemia, calcific atheroma, gout, and renal lithiasis [11-13]. Pulp stones have recently been linked to several systemic illnesses, including autoimmune diseases, diabetes mellitus (DM), renal diseases, and coronary artery disease (CAD). Renal calculi and pulp stones are significantly correlated by a small number of authors [9]. Furthermore, scientists contemplated that pulp stone detection may serve as a systemic disease diagnostic marker [14].

In the present day, cardiovascular disease (CVD) is a leading cause of morbidity and death. The main cause of CAD, which results in ischemic heart disease, is atherosclerosis. Numerous writers have discovered a link between pulp stones and CVD [14,15]. Pulp stones and hypertension (HTN) have recently been connected [9]. It is also known that systemic illnesses can upset the delicate balance between the mouth and microbial flora, which can increase pathogenic bacteria [16]. The connection between pulp stones and systemic disease has not been thoroughly investigated in this part of the Eastern region of Saudi Arabia. Considering the aforementioned fact, the purpose of our study was to assess the prevalence of pulp stones, DM, and cardiovascular illnesses in Saudi Arabian patients.

Thus, subsequently, routine dental radiographs can be extremely helpful as a swift and non-invasive diagnostic tool for early detection of any possible systemic diseases, further heralding an era of interdisciplinary partnership for providing better healthcare. This present research aimed to estimate the pulp stones prevalence by orthopantomographs (OPGs) and to assess its association with gender, nationality, age, dental status, and systemic diseases.

## Materials and methods

The Research Committee of the College of Dentistry at Imam Abdulrahman Bin Faisal University in Saudi Arabia gave ethical permission for the study (EA:2019014). The study examined the radiographs and medical records of patients who visited the Department of Oral Diagnosis and Screening clinics at the university's College of Dentistry from January 2017 to June 2018.

Patients were divided into Saudi and non-Saudi categories based on their nationality. The inclusion criteria included patients who received dental treatment at Imam Abdulrahman Bin Faisal University Dental Hospital between January 2017 and June 2018, had a complete set of OPGs available, and had no history of dental trauma, endodontic treatment, or orthodontic intervention. Patients of all ages were included in the study. Exclusion criteria included poor-quality radiographs and incomplete medical records.

**Figure 1:**
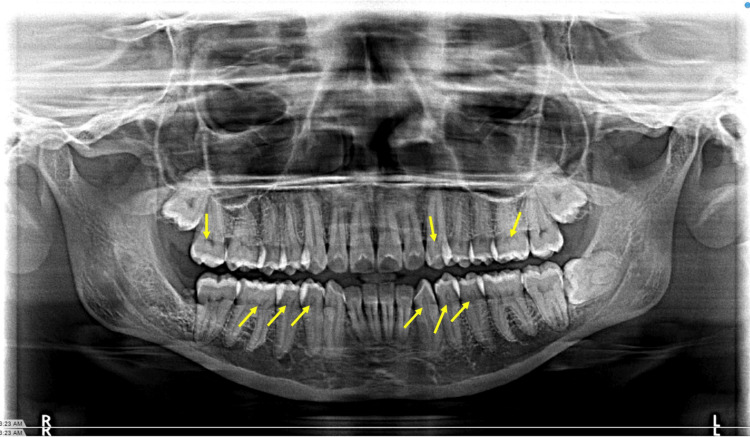
Sample OPG showing pulp stones in maxillary and mandibular teeth. Yellow arrow pointing at the pulp stone. OPG: orthopantomographs

Based on previously published literature and the expected prevalence of pulp stones, the sample size was calculated to include a minimum of 10 patients per age group to achieve statistical power. The data on gender, nationality, medical condition, and dental status were collected from the medical records, along with the prevalence of pulp stones in both the maxillary and mandibular arches (Figure 1).

Two trained and calibrated radiologists with expertise in detecting anatomical dental landmarks independently reviewed the OPGs to identify the presence of pulp stones and then compared their findings, resolving any discrepancies through discussion to reach a consensus. Pulp stone prevalence was recorded for different age groups, categorized as follows: Group 1 (5-15 years), Group 2 (16-25 years), Group 3 (26-35 years), Group 4 (36-45 years), Group 5 (46-55 years), Group 6 (56-65 years), and Group 7 (65+ years). Statistical Product and Service Solutions (SPSS, version 22; IBM SPSS Statistics for Windows, Armonk, NY) was used for statistical analysis. A chi-square test was conducted to analyze the association between variables (systemic diseases), including gender, nationality, and age, in participants with and without pulp stones. Logistic regression analysis was performed to assess the relationship between the presence of pulp stones and potential predictors, including age, gender, diabetes, blood pressure, and the presence of systemic diseases. A result of p<0.05 was considered as statistically significant.

## Results

The distribution of patients according to gender, nationality, and age is mentioned in Table 1. A total of 153 patient records were examined, which included 51% (n=78) male and 49% (n=75) female patients. The radiographic evaluation revealed that the pulp stones were detected in 43.1% (n=66) of the total patients, with 47% (n=31) in males and 53% (n=35) in females. Among the nationalities, it was shown that Saudi patients were the most affected, with 57.6% (n=38), while 42.4% (n=28) were non-Saudi, of the total patients having pulp stones. The maximum pulp stone prevalence was observed in age Group 4 with 21.2% (n=14), followed by age Group 6 with 19.7% (n=13). On the other hand, the minimum prevalence was observed in age Group 2 with 7.6% (n=5).

**Table 1:** Prevalence (%) of pulp stones according to demographics. Statistically significant difference (p<0.05)

		With pulp stones (%) (n=66)	Without pulp stones (%) (n=87)	Total (%)	P-Value
Gender					
Males		31 (47%)	47 (54%)	78 (51%)	0.387
Females		35 (53%)	40 (46%)	75 (49%)	
Total		66 (43.1%)	87 (56.9%)	153 (100%)	
Nationality					
Saudi		38 (57.6%)	60 (69%)	98 (64.1%)	0.146
Non-Saudi		28 (42.4)	27 (31%)	55 (35.9%)	
Total		66 (43.1%)	87 (56.9%)	153 (100%)	
Age (years)					
Group 1 (5-15)		6 (9.1%)	57 (65.5%)	63 (41.2%)	0.387
Group 2 (16-25)		5 (7.6%)	5 (5.7%)	10 (6.5%)	
Group 3 (26-35)		11 (16.7%)	5 (5.7%)	16 (10.5%)	
Group 4 (36-45)		14 (21.2%)	7 (8.0%)	21 (13.7%)	
Group 5 (46-55)		11 (16.7%)	7 (8.0%)	18 (11.8%)	
Group 6 (56-65)		13 (19.7%)	4 (4.6%)	17 (11.1%)	
Group 7 (65+)		6 (9.1%)	2 (2.3%)	8 (5.2%)	
Total		66 (43.1%)	87 (56.9%)	153 (100%)	

The distribution of teeth with pulp stones based on the arch and tooth type is listed in Table 2. The pulp stones were more prevalent in teeth of the maxillary arch with 79.1% (n=155) out of all teeth (n=196) detected with pulp stones, while the teeth in the mandibular arch had 20.9% (n=41) prevalence of pulp stones. Additionally, for teeth with pulp stones, first molars showed the highest occurrence with 41.3% (n=81), followed by second molars with 37.8% (n=74), third molars with 10.7% (n=21), and second premolars with 5.1% (n=10). In contrast, the first premolars, central incisors, lateral incisors, and canines presented a low incidence of 2.6% (n=5), 1% (n=2), 0.5% (n=1), and 0.5% (n=1), respectively. In the maxillary arch, the most affected tooth was the first molar, which contributed up to 35.7% (n=70), followed by the second molar with 30.6% (n=60). On the other hand, the most affected tooth in the mandibular arch was the second molar with 7.1% (n=14), followed by the first molar with 5.6% (n=11).

**Table 2:** Prevalence (%) of pulp stones according to arch and tooth type.

Tooth type	Maxillary teeth with pulp stones	Mandibular teeth with pulp stones	Total
Third Molar	21 (10.7%)	0 (0%)	21 (10.7%)
Second Molar	60 (30.6%)	14 (7.1%)	74 (37.8%)
First Molar	70 (35.7%)	11 (5.6%)	81 (41.3%)
Second Premolar	10 (5.1%)	0 (0%)	10 (5.1%)
First Premolar	5 (2.6%)	0 (0%)	5 (2.6%)
Canine	1 (0.5%)	0 (0%)	1 (0.5%)
Lateral Incisor	1 (0.5%)	0 (0%)	1 (0.5%)
Central Incisor	2 (1%)	0 (0%)	2 (1%)
Total	155 (79.1%)	41 (20.9%)	196 (100%)

The distribution of medically compromised patients and the prevalence of pulp stones according to the medical conditions are shown in Table 3. Out of all examined patients, 30.1% (n=46) were medically compromised. In these medically compromised patients, the radiographic examination revealed that 56.5% (n=26) had pulp stones.

**Table 3:** Prevalence (%) of pulp stones according to systemic diseases. ^1 ^Allery, ulcers, epilepsy, G6DP, liver disorder * showing significant difference (p<0.05)

Systemic diseases	With pulp stones (%) (n=66)	Without pulp stones (%) (n=87)	P-Value
Hypertension	12 (18.2%)	3 (3.4%)	0.004*
Diabetic Patients	11 (16.7%)	3 (3.4%)	0.009*
Rheumatoid arthritis	9 (13.6%)	5 (5.7%)	0.094
Sensorineural hearing loss	4 (6.1%)	3 (3.4%)	0.463
Sickle cell anemia	4 (6.0%)	10 (11.5%)	0.275
Asthma	3 (4.5%)	1 (1.1%)	0.316
Hypoparathyroidism	2 (3.0%)	0 (0.0%)	0.185
Renal Disorder	0 (0.0%)	2 (2.3%)	0.506
Other Medical Conditions^1^	7 (10.6%)	5 (5.7%)	0.268

A logistic regression analysis (Table 4) was conducted to assess the association between various factors and the presence of pulp stones. Age demonstrated a statistically significant association with pulp stones (p=0.001). The odds ratio (OR) for age was 1.047, with a 95% confidence interval (CI) ranging from 1.025 to 1.070, indicating that each additional year of age increases the odds of having pulp stones. Gender showed no statistically significant association with pulp stones (p=0.546), with an OR of 1.308 (95% CI: 0.548-3.120). For diabetes, the OR was 2.061, with a 95% CI ranging from 0.540 to 7.872 (p=0.290), which shows that individuals with diabetes have over twice the odds of having pulp stones compared to those without diabetes; however, this result was not statistically significant. Hypertension did not show a statistically significant association in the logistic regression model (p=0.185), with an OR of 0.473 (95% CI: 0.157-1.430). Other medical conditions (allergy, ulcers, epilepsy, G6DP, liver disorder) showed an OR of 1.694 (95% CI: 0.242-11.995) and a p-value of 0.498, indicating no significant relationship between these conditions and pulp stones.

**Table 4:** Logistic regression analysis of factors associated with the presence of pulp stones. * showing aa significant difference (p<0.05) ^_1_^ Allery, ulcers, epilepsy, G6DP, liver disorder

Variables	Odds ratio	95% C.I. for odds ratio		P-value
		Lower	Upper	
Age	1.047	1.025	1.070	.001*
Gender	1.308	.548	3.120	.546
Diabetes	2.061	.540	7.872	.290
Hypertension	0.473	0.157	1.43	0.185
Other Medical Conditions^1^	1.694	0.242	1.995	0.498

## Discussion

In this study, the OPG of each patient was retrieved and analyzed by two trained and calibrated independent oral and maxillofacial radiologists for the prevalence of pulp stones. The prevalence of pulp stone in this study was 43.1%, and the content of pulp stone was higher in females than in males. In contrast, 51% of pulp stones were recorded by Kannan et al., and there was no discernible relationship between gender and ethnic race [4]. Further, a similar higher prevalence (50.9%) was noted in a study from Saudi Arabia [17]. Compared to the population of Saudi Arabia in our current comparative study, the aforementioned observations were more common. Nevertheless, research by Al-Nazhan et al. in the Saudi Arabian population found an astoundingly low (10.2%) frequency [18]. These differences in prevalence could be due to the different sample sizes. These differences in prevalence could be due to the various sample sizes and may also be attributed to the geneto-regional variations.

According to previous studies, the teeth in the maxillary arch had greater pulp stone prevalence compared to the teeth in the mandibular arch [19-21]. In this current study, the pulp stone prevalence was higher in the maxillary first molar, followed by the maxillary second molar and this agrees with other studies [20-22]. A potential reason could be that the first molars erupt early, subjecting them to more degenerative changes over a long period, validating the findings that calcification of the pulp increases over time [22]. In addition, the same study found that the most plausible explanation is that, as the pulp ages, its structure undergoes significant changes, including an increase in fibrous materials and mucopolysaccharides, along with a marked decrease in pulp cells [22]. These factors collectively contribute to calcification. Moreover, the maximum chewing force is exerted on the molars, which may further promote calcification. This likely explains the higher prevalence of pulp stones observed in patients in their fourth to sixth decades of life (Groups 4-6). The regression analysis for our study shows that age has a statistically significant association with pulp stones (p=0.001). This finding is consistent with observations by Lyngdoh et al. [21] and Patil et al. [17]. Similarly, Al-Naznan et al. [18] noted an increased occurrence of pulp stones in patients over 55 years of age. In contrast, studies by Turkal et al. [23] and Deniz et al. [24] reported no significant correlation between age and pulp stone occurrence.

Geneto-systemic disorders, such as cardiovascular, nephrotic, endocrine, gallbladder, pancreatic, hepatic, etc., have become common and have increased expressively high over the last decade [25-27]. In 2005, Edds et al. reported that 74% of patients with known cardiovascular disease had observable pulp stones, while only 39% of patients with no history of cardiovascular disease reported pulp stones [9]. Sayegh et al. concluded that systemic disorders such as arteriosclerosis and renal lithiasis can be regarded as predisposing factors for calcification of the pulp [28]. In our study, 12 out of 15 patients with hypertension (80%) had pulp stones, and univariate analysis revealed a statistically significant association between hypertension and the presence of pulp stones (p=0.004), suggesting a potential link between these conditions. However, this association did not remain significant in the regression analysis (p=0.185), where other variables were accounted for. This finding is nearly identical to that reported by Nachiappan et al. [14], but in contrast to the results of Bains et al. [6]. Hypertension is a well-considered risk factor in atherosclerosis, the molecular and cellular causes and the impact of hypertension are more precisely defined, and these two processes have some similar mechanisms [29-31].

Out of two renal disorder patients, none (0%) had pulp stones. A study conducted by Stafne et al. reported that pulp stones are not directly responsible for the development of renal and gall stones [31]. That being said, da Costa et al. hypothesized that nanobacteria could lead to pulp, kidney, and gallstone formation [32]. Meanwhile, of the 14 patients with rheumatoid arthritis, the frequency of pulp stones was reported in nine patients (64.3%), which was unswerving with the findings of Chen et al., where three of four (75%) rheumatoid arthritis patients exhibited pulp stones [33].

In the present study, a greater prevalence of pulp stones in patients with DM (78.6%) was reported, with 11 patients out of 14 patients presenting with pulp stones. Univariate analysis revealed a statistically significant association between DM and the presence of pulp stones (p=0.009). However, this association did not remain significant in multivariate regression analysis (p=0.290). A comparable study by Nayak et al. reported pulp stones in 18% of DM patients [5]. Similarly, Bender et al. histologically analyzed human pulp in non-carious teeth collected from seven patients with long-term diabetes [34]. It was concluded that angiopathic calcification and the thickening of the basement membrane were found in both small and large blood vessels and that changes in vascularity tend to be more prominent in the pulpal core [34].

Hypoparathyroidism is a metabolic condition resulting from a deficiency or absence of parathyroid hormone secretion, characterized by low serum calcium and elevated serum phosphorus levels. Common features in patients with hypoparathyroidism include widened pulp chambers and dental pulp calcifications. In the present study, 100% of the hypoparathyroid patients (two out of two) exhibited pulp stones. However, we were unable to correlate this finding with existing literature due to a lack of available studies on the topic.

Further, one of the astonishing findings in the study is that of sensorineural hearing loss, where four of seven (57.1%) sensorineural hearing loss patients exhibited pulp stones. Sensory hearing loss is caused by abnormal structure or calcification of the hair cells of the organ of Corti in the cochlea. Additional complete studies are needed to verify the findings.

There are various blood disorders reported in the medical field that have oral cavity-related manifestations [35]. One of these blood disorders is anemia, which can be defined as the deficiency of hemoglobin or red blood cells, leading to inadequate delivery of oxygen to the tissues [36]. Moreover, one of the commonly inherited types is sickle cell anemia, which occurs due to the alteration in the structure and morphology of the red blood cells caused by the hemoglobin gene [37]. Pulp stones are regarded as one of the main dental manifestations of sickle cell anemia [38,39]. Correspondingly, the current study disclosed that four of 14 (29%) sickle cell anemia patients exhibited pulp stones. Unfortunately, we could not correlate this finding with the other studies due to a lack of available medical literature on the topic.

Asthma is an inflammatory medical condition of the lungs characterized by the constriction of the bronchioles, leading to breathing difficulties and coughing [40]. The relationship between asthma and oral health is a topic of debate in dentistry. In the present study, a higher pulp stones prevalence was observed in patients with asthma, with three out of 14 (75%) patients presenting with pulp stones.

The results presented in this study may not match those found in earlier medical publications. There are several possible reasons for this mismatch, including the sample size, age, ethnicity, and methodology used. The tiny sample size of this study was one of its limitations. A larger sample size could have allowed for a more comprehensive analysis of the relationship between pulp stone formation and systemic diseases. Other drawbacks were the failure to examine the entire and intricate structure of pulp stones and the neglect of additional factors that contribute to pulp irritation, such as periodontal diseases.

Accounting for confounding variables is critical to understanding whether systemic conditions directly influence pulp stone formation or whether their association is mediated by other factors. While univariate analysis can reveal initial associations, multivariate models are necessary to adjust for potential confounders such as age, gender, or other medical conditions. Based on the findings, when confounding variables are taken into account, there is no significant difference in developing systemic illness for patients with pulp stones. Therefore, screening for these patients is necessary to determine the severity of these consequences early on. Thus, it is critical to promote the use of dental radiographs in the identification of individuals with systemic diseases to facilitate additional screening and assessment.

This research has certain limitations, such as including only patients who are seeking dental treatment at Imam Abdulrahman Bin Faisal University, which could undermine the true correlation between the types and severity of medical problems. Additionally, other systemic diseases have not been considered such as parathyroid abnormalities and conditions affecting the bones, kidneys, joints, and gastrointestinal tract. Furthermore, the superimposition of structures and distortion of images in two-dimensional radiography may compromise the accuracy of the results. However, a study design like this provides insight that helps determine how various inflammatory oral diseases and pulp calcification are related. The true number of pulp stones may be slightly higher than what is shown on the radiographs because a histological section is not taken. To determine the link between the factors examined in this study, a prospective cohort study should be planned, particularly regarding pulp stones associated with long-term inflammatory oral diseases.

## Conclusions

In conclusion, this study suggests that, when confounding variables are taken into account, patients with pulp stones may not be at an increased risk of developing systemic diseases. This research represents the first investigation of its kind conducted in the Eastern Province of Saudi Arabia, providing preliminary data on the utility of dental radiographs for detecting pulp stones and their implications for endodontic therapy. Furthermore, our findings support the potential of dental radiographs as a rapid screening tool for the early identification of possible systemic diseases. Further research is warranted to explore these associations and enhance clinical practices.
